# Genome-Wide SNP Linkage Mapping and QTL Analysis for Fiber Quality and Yield Traits in the Upland Cotton Recombinant Inbred Lines Population

**DOI:** 10.3389/fpls.2016.01356

**Published:** 2016-09-08

**Authors:** Cong Li, Yating Dong, Tianlun Zhao, Ling Li, Cheng Li, En Yu, Lei Mei, M. K. Daud, Qiuling He, Jinhong Chen, Shuijin Zhu

**Affiliations:** ^1^Department of Agronomy, Zhejiang UniversityHangzhou, China; ^2^Department of Biotechnology and Genetic Engineering, Kohat University of Science and TechnologyKohat, Pakistan

**Keywords:** upland cotton, cotton 63K SNP array, linkage analysis, molecular marker, QTLs

## Abstract

It is of significance to discover genes related to fiber quality and yield traits and tightly linked markers for marker-assisted selection (MAS) in cotton breeding. In this study, 188 F_8_ recombinant inbred lines (RILs), derived from a intraspecific cross between HS46 and MARCABUCAG8US-1-88 were genotyped by the cotton 63K single nucleotide polymorphism (SNP) assay. Field trials were conducted in Sanya, Hainan Province, during the 2014–2015 cropping seasons under standard conditions. Results revealed significant differences (*P* < 0.05) among RILs, environments and replications for fiber quality and yield traits. Broad-sense heritabilities of all traits including fiber length, fiber uniformity, micronaire, fiber elongation, fiber strength, boll weight, and lint percentage ranged from 0.26 to 0.66. A 1784.28 cM (centimorgans) linkage map, harboring 2618 polymorphic SNP markers, was constructed, which had 0.68 cM per marker density. Seventy-one quantitative trait locus (QTLs) for fiber quality and yield traits were detected on 21 chromosomes, explaining 4.70∼32.28% phenotypic variance, in which 16 were identified as stable QTLs across two environments. Meanwhile, 12 certain regions were investigated to be involved in the control of one (hotspot) or more (cluster) traits, mainly focused on Chr05, Chr09, Chr10, Chr14, Chr19, and Chr20. Nineteen pairs of epistatic QTLs (e-QTLs) were identified, of which two pairs involved in two additive QTLs. These additive QTLs, e-QTLs, and QTL clusters were tightly linked to SNP markers, which may serve as target regions for map-based cloning, gene discovery, and MAS in cotton breeding.

## Introduction

The *Gossypium* genus is the most important source of natural textile fiber. It consists of 50 species approximately, including four cultivated species, *G. arboreum, G. herbaceum, G. hirsutum*, and *G. barbadense*. *G. hirsutum*, accounting for 95% of overall cotton production, is characterized by high yield, moderate fiber quality, and wide adaptability (Cai et al., 2014). In the past few years, area under cotton cultivation has declined worldwide, mainly due to high production costs and strong market competition with other crops (Mei et al., 2013). Thus, developing new cotton cultivars with superior fiber quality and high yields are immense to meet the demand of textile industry demand and to maintain the profitability of cotton for its growers.

Fiber quality and yield traits are complex traits, controlled by a multitude of quantitative trait locus (QTLs; Said et al., 2013). There is a complicated genetic correlation between fiber quality and yield due to different population types and parental lines (Qin et al., 2008; Yu et al., 2013; Zhang et al., 2014; Cao et al., 2015; Wang et al., 2015a). Therefore, improving yield and fiber quality, simultaneously, is a long-term challenge for cotton breeders. Traditional breeding procedures are increasingly difficult because of long duration and low selective efficiency (Shen et al., 2005). Marker-assisted selection (MAS) could be one of the more efficient approaches for breeding elite upland cotton cultivars. To date, majority of cotton genetic maps have been developed based on interspecific populations (Reinisch et al., 1994; Lacape et al., 2003; Nguyen et al., 2004; Rong et al., 2004; Guo et al., 2007; Yu et al., 2011b, 2013), which had little usage in upland cotton breeding programs (Ulloa et al., 2002). The intraspecific genetic maps of upland cotton have been constructed to detect QTLs for fiber quality and yield traits (Zhang et al., 2003, 2009; Shen et al., 2006; Wang et al., 2006, 2015b; Qin et al., 2008; Wu et al., 2008; Sun et al., 2011; Liang et al., 2013; Tan et al., 2014; Islam et al., 2015; Zhang Z. et al., 2015). However, due to low levels of intraspecific DNA marker polymorphisms in upland cotton, most intraspecific maps had a relatively low density and could not satisfy for MAS and map-based cloning. Therefore, it is necessary to develop new type of markers which can enable mapping upland cotton populations to obtain high polymorphism.

In comparison with other molecular markers, single nucleotide polymorphisms (SNPs) marker which provides the most abundant form of genetic variations, and is characterized by lower mutation rates, higher numbers, and higher accuracy (Ball et al., 2010; Yu et al., 2011a). Previous research has showed that SNPs plays role in phenotypic changes and act as functional marker for traits in MAS, when located in a gene or promoter region (Beales et al., 2005; Konishi et al., 2006). It led to the discovery of superior high-density SNP gene-chip technology was then developed as a superior method for linkage mapping and QTL detection. Now, it is being used extensively to detect QTL in bi-parental populations of many crop species (McNally et al., 2009; Gao et al., 2015). But only a few studies had reported its use in intraspecific populations of upland cotton, either with a limited number (Byers et al., 2012; Yu et al., 2012; Gore et al., 2014; Zhu et al., 2014) or with lower density (Hulse-Kemp et al., 2015; Wang Y. et al., 2015). So, there is a huge knowledge gap to be filled by a comprehensive study.

Herein, a 63 K Illumina SNP assay was used to screen 188 recombinant inbred lines (RILs) derived from the cross of HS46/MARCABUCAG8US-1-88, and a final map with 2618 loci and 0.68 cM high density map was constructed. The aims of this study were to identifying stable QTLs for fiber quality and yield traits and their tightly linked SNP markers for MAS in upland cotton breeding.

## Materials and Methods

### Plant Materials

A RIL population of 188 individual lines was developed following a modified single-hill procedure (bulked progeny row; Wu et al., 2008) by crossing two upland cotton cultivars, HS46 and MARCABUCAG8US-1-88. The former is a commercial cultivar with good fiber qualities and higher yield, and the later, a male parent was a germplasm with good resistance. The RILs and their parents were kindly provided by USDA-ARS, Starkville, MS, USA in 1999 (Liu et al., 2012).

In September 2014, the seeds of 188 RILs and two parents were sown in Yacheng and Baogang (two different environments were hereinafter referred to as Yc and Bg, respectively) in Sanya, Hainan Province, China. A completely randomized block design with two replications was applied in each location. Plot size was one row with 7.0 m long and 0.8 m wide. Standard cultivation, weed and insect control practices were followed throughout the growing season.

### Phenotypic Measurement and Analysis

A total of 20 normally open bolls were hand-harvested from each line. Approximately 20 g of fiber from each sample was measured by HVI 1000 (Uster^®^Hvispectrum, Spinlab, USA) under controlled environmental conditions (20°C and 65% RH) in the Cotton Quality Supervision, Inspection and Testing Center, Ministry of Agriculture, Anyang, Henan province, China. The fiber quality traits include fiber length (FL, mm), fiber length uniformity (FU, %), micronaire (MIC), fiber elongation (FE, %), and fiber strength (FS, cN.tex^-1^). Yield traits consist of boll weight (BW, g) and lint percent (LP, %).

The basic statistics for the phenotypic data of the RILs, the significance of differences for each trait between the two parents, and the correlation among different traits were calculated by SPSS20.0. The variance components for fiber quality and yield traits were estimated by QTModel^1^.

### SNP Maker Analysis and Genotyping

Genomic DNA was extracted from young leaves of the 188 RILs and two parents using modified CTAB method (Paterson et al., 1993).

The 188 RILs and their parents were genotyped with cotton 63K SNP array (Hulse-Kemp et al., 2015) from Emei Tongde Technology Development, Co. Ltd (EMTD; Beijing, China^2^). The array, consisted of 63,058 SNPs, were derived from published literatures (Van Deynze et al., 2009; Byers et al., 2012; Lacape et al., 2012; Rai et al., 2013). Candidate SNPs suitable for further analysis were identified as follows: (1) SNPs were filtered by excluding those with monomorphic markers or with poor quality data; (2) SNPs which the parental genotypes were inconsistent with progeny genotypic ratios or parental genotypes data had missing information were removed from the dataset; (3) SNPs of 188 RILs with missing values more than 40% were removed.

Candidate SNPs, obtained from the array, were further aligned to the tetraploid upland cotton (TM-1) reference genome (Zhang T. et al., 2015), using BWA software (Li and Durbin, 2009). Only SNPs with less than two mismatches based on a high quality sequence and a mapping Q-Value > 20 were used. The retained SNPs were sent to samtools (Li et al., 2009) and then screened for polymorphism between the mapping parents. Polymorphic SNPs were classified based on Illumina GenTrain score and call frequencies across samples (Hulse-Kemp et al., 2015). Minor allele frequencies of polymorphic markers were only used to genotype 188 RILs.

### Map Construction

Linkage maps were constructed by JoinMap 4.0 Version Software (Van Ooijen, 2006), using a regression approach with the log of odds (LODs) score of 3∼10 and the jump threshold of 0.5. Converting recombination frequencies into map distances were calculated using Kosambi’s mapping function (Kosambi, 1944).

The chi-square analysis was performed to test segregating markers which deviated from 1:1 expected segregation ratio. A segregation distorted region (SDR) was defined as region with at least three adjacent loci showing significant segregation distortion (*P* < 0.05; Yu et al., 2011b).

Based on the results of SNPs aligned to the *G. hirsutum* reference genome by BWA (Li and Durbin, 2009), software CIRCOS 0.69 was used to compare the collinearity of SNPs based on their genetic positions and physical positions.

### QTL Analysis

Fiber quality and yield traits related QTLs detection were performed by WinQTLCart2.5 software (Wang et al., 2001), using composite interval mapping (CIM) approach. The LOD threshold of significant QTL was calculated by 1,000 permutation tests with a significance level of *P* < 0.05, a mapping step of 1.0 cM, and five control markers. LOD score values between 2.5 and permutation test LOD threshold were used to declare suggestive QTL. E-QTLs were detected by IciMapping ver. 4.0 software (Li et al., 2007) using multi-environment trials (METs) function and the inclusive composite interval mapping (ICIM) method. The e-QTLs identification was done with pre-adjusted IciMapping parameters: Scan = 5 cM, LOD = 5.0, and PIN = 0.0001. A graphical representation of the linkage groups and QTLs was created by Map Chart 2.2 (Voorrips, 2002).

QTLs were named as following: q + trait abbreviation+ chromosome number+ QTL number.

## Results

### Phenotypic Evaluation of RIL Populations

Descriptive statistics for the fiber quality and yield traits of the RIL population, as well as their parents across two environments were presented in **Table 1**. FL, FU, BW, FS, and MIC of HS46 showed significantly higher than those of MARCABUCAG8US-1-88 in one or both locations; however, there were no significant differences were found for FE and LP between parental lines. In the RIL population, all analyzed traits presented continuous variation and transgressive segregation, and accorded with normal distributions.

**Table 1:** Phenotypic variation of five fiber quality traits and two yield traits for the upland cotton RILs and their parents.

Traits^a^	Environment^b^	Parents			RILs					
		HS46(P1)	MAR(P2)	P1–P2	Mean	*SD*	Skewness	Kurtosis	Minimum	Maximum
FL	Yc	31.00	29.84	1.17^∗∗^	30.49	1.15	0.18	0.35	27.26	34.12
	Bg	30.47	29.17	1.31^∗∗^	30.21	1.02	0.06	0.16	27.20	33.39
FU	Yc	86.35	85.27	1.08^∗∗^	85.57	0.83	0.28	0.17	83.60	88.50
	Bg	85.92	84.90	1.02^∗^	85.39	0.86	-0.30	-0.38	83.10	87.30
MIC	Yc	4.12	3.77	0.36^∗^	3.83	0.32	0.20	0.14	3.06	4.76
	Bg	3.88	3.75	0.13	3.50	0.39	0.21	-0.29	2.68	4.54
FE	Yc	6.43	6.52	-0.08	6.38	0.70	0.08	-0.40	4.90	8.20
	Bg	5.98	6.07	-0.09	5.76	0.66	0.33	0.14	4.10	8.10
FS	Yc	30.09	28.09	2.01^∗∗^	29.92	1.66	0.48	0.09	26.61	35.67
	Bg	29.45	28.67	0.78	29.67	1.59	0.05	0.28	25.13	34.11
BW	Yc	5.43	4.91	0.51^∗∗^	5.37	0.56	0.47	0.61	3.96	7.09
	Bg	5.81	5.37	0.44^∗^	5.60	0.65	-0.18	0.01	3.92	7.41
LP	Yc	37.07	38.74	-1.67	37.73	1.94	0.22	0.63	31.63	43.87
	Bg	39.07	39.87	-0.81	38.16	1.75	-0.47	0.71	31.69	42.15

*^∗,∗∗^Significant at *P* = 0.05 and *P* = 0.01, respectively. ^a^FL, fiber length; FU, fiber uniformity; MIC, micronaire; FE, fiber elongation; FS, fiber strength; BW, boll weight; LP, lint percentage. ^b^Yc, Yacheng of Hainan Province; Bg, Baogang of Hainan Province.*

Results showed that the FL had the highest broad-sense heritability among all traits, indicating that it could be mainly controlled by genotype, while remaining six traits had lower broad-sense heritability with values of 0.26 for FU, 0.27 for MIC, 0.37 for FE, 0.28 for FS, 0.31 for BW, and 0.38 for LP, suggesting the environmental effects were important for the performance of these traits (**Table 2**).

**Table 2:** Analysis of variance (ANOVA) for five fiber quality traits and two yield traits in the upland cotton RIL population across two environments.

Factor^b^	DF	Sum of squares						
		FL^a^	FU^a^	MIC^a^	FE^a^	FS^a^	BW^a^	LP^a^
Gen	187	803.03ˆ**	373.93ˆ**	77.42ˆ**	288.82ˆ**	1437.08ˆ**	199.49ˆ**	1953.4ˆ**
Env	1	14.82ˆ**	6.22ˆ**	20.48ˆ**	70.59ˆ**	11.45	12.34ˆ**	38.72ˆ**
Gen^∗^Env	187	82.57	157.72	17.31	58.16	539.28	62.06	522.32
Rep	1	0.27	0.87	8.8ˆ**	4.57ˆ**	0.36	5.64ˆ**	5.88
Residual	375	172.19	304.43	54.00	126.38	1140.54	139.02	1108.54
Broad-sense heritability		0.66	0.26	0.27	0.37	0.28	0.31	0.38

*^∗,∗∗^Significant at *P* = 0.05 and *P* = 0.01, respectively. ^a^FL, fiber length; FU, fiber uniformity; MIC, micronaire; FE, fiber elongation; FS, fiber strength; BW, boll weight; LP, lint percentage. ^b^Gen, genotype; Env, environment; rep, replication.*

The correlation analysis for fiber and yield traits based on RILs data over two environments (**Table 3**). Results revealed significant negative correlation of LP with FS and FU, and significant positive correlation with MIC and FE. Similarly, BW was significant positively correlated with MIC and FS. Among the five fiber traits, all trait pairs presented significantly correlation except for FU – FE, FU – MIC, and FS – FE.

**Table 3:** Correlation coefficients among all traits involved in fiber quality and yield in the upland cotton RIL population across two environments.

Traits^a^	FL	FU	MIC	FE	FS	BW	LP
FL	1						
FU	0.56^∗∗^	1					
MIC	-0.30^∗∗^	-0.04	1				
FE	-0.26^∗∗^	0.00	0.41^∗∗^	1			
FS	0.17^∗∗^	0.24^∗∗^	0.15^∗∗^	0.01	1		
BW	-0.02	0.05	0.38^∗∗^	0.03	0.17^∗∗^	1	
LP	-0.18	-0.14^∗∗^	0.09^∗∗^	0.14^∗∗^	-0.18^∗∗^	-0.07	1

*^∗,∗∗^Significant at *P* = 0.05 and *P* = 0.01, respectively. ^a^FL, fiber length; FU, fiber uniformity; MIC, micronaire; FE, fiber elongation; FS, fiber strength; BW, boll weight; LP, lint percentage.*

### Map Construction

**FIGURE 1:**
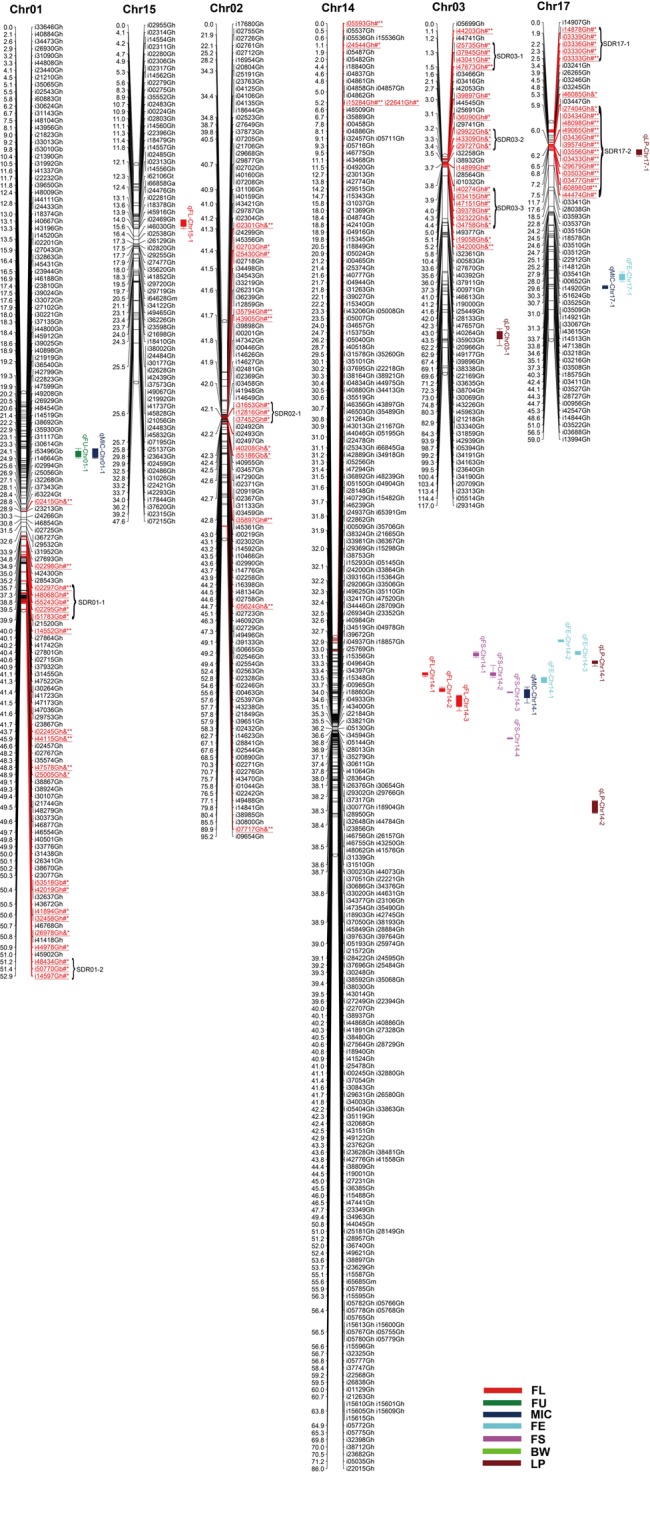
**Genetic maps of Chr01/Chr15, Chr02/Chr14, and Chr03/Chr17 homoeologous chromosomes and QTL detection for fiber quality and yield traits in RIL population.** Map distances were given in centimorgans (cM). Markers showing segregation distortion are underlined and indicated by red color and asterisks (^∗^*P* < 0.05; ^∗∗^*P* < 0.01). Number sign (#) markers represent skewing toward the HS46 allele, ampersand (&) markers represent skewing toward the MARCABUCAG8US-1-88 allele. Segregation distortion regions (SDRs) are named as ‘Chromosome + No. SDR,’ for example, SDR01-1 refers to the first SDR on Chr01. Solid bars with different colors represent different traits and the legend is given at the end of figure. FL, fiber length; FU, fiber uniformity; MIC, micronaire; FE, fiber elongation; FS, fiber strength; BW, boll weight; LP, lint percentage.

**FIGURE 2:**
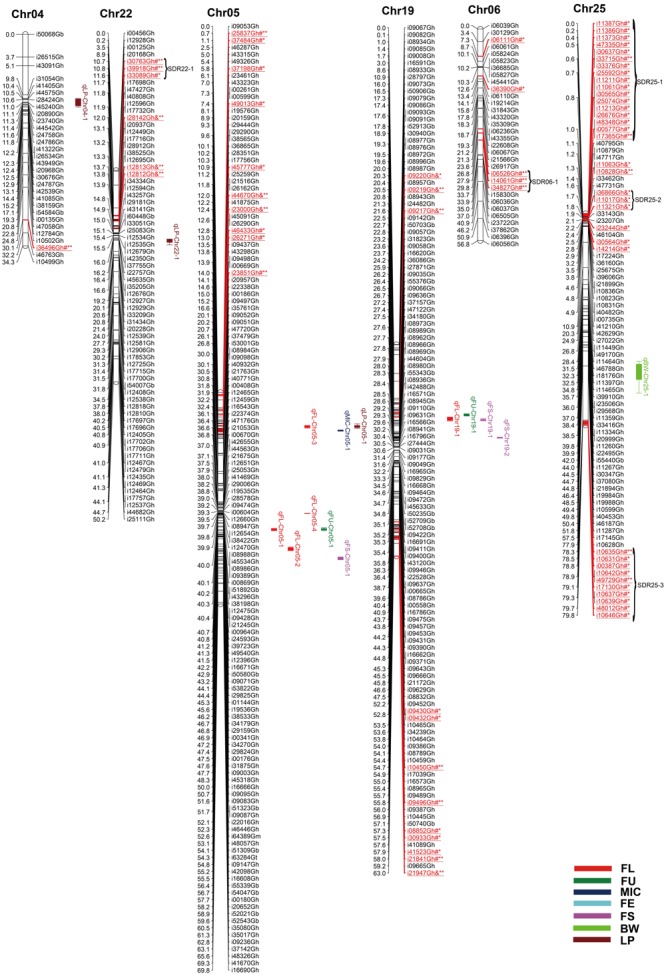
**Genetic maps of Chr04/Chr22, Chr05/Chr19 and Chr06/Chr25 homoeologous chromosomes and QTL detection for fiber quality and yield traits in RIL population.** All legends are same as described for **Figure 1**.

**FIGURE 3:**
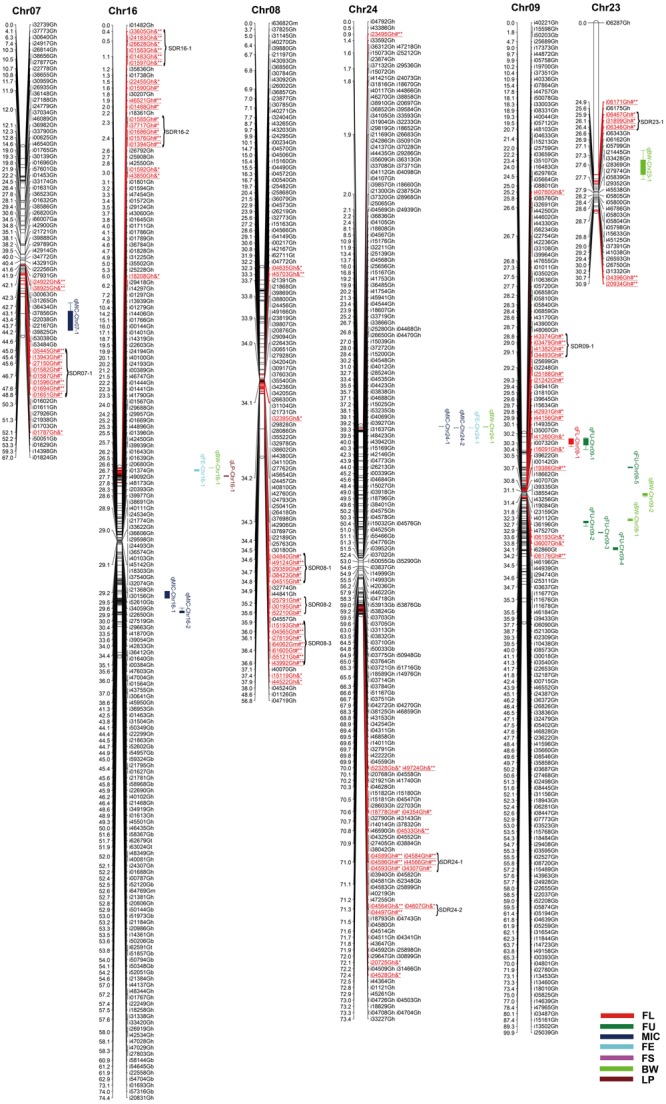
**Genetic maps of Chr07/Chr16, Chr08/Chr24, and Chr09/Chr23 homoeologous chromosomes and QTL detection for fiber quality and yield traits in RIL population.** All legends are same as described for **Figure 1**.

**FIGURE 4:**
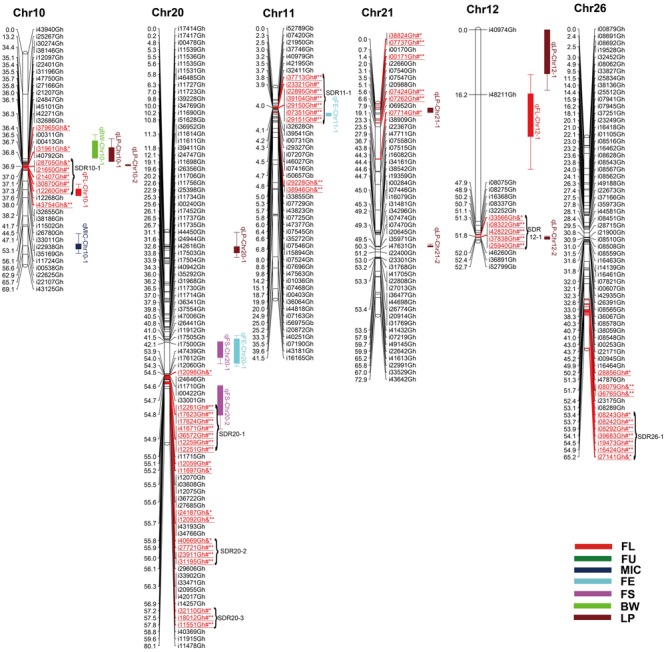
**Genetic maps of Chr10/Chr20, Chr11/Chr21, and Chr12/Chr26 homoeologous chromosomes and QTL detection for fiber quality and yield traits in RIL population.** All legends are same as described for **Figure 1**.

**FIGURE 5:**
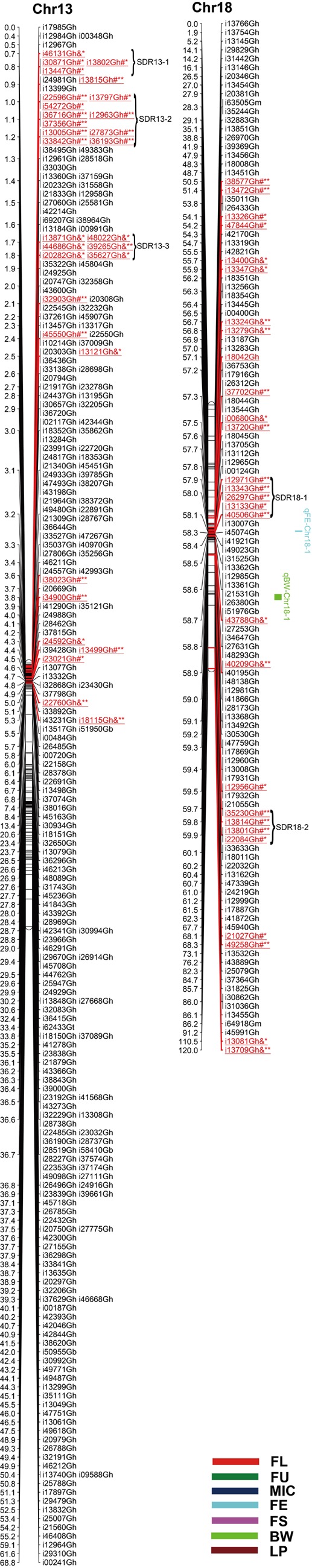
**Genetic maps of Chr13/Chr18 homoeologous chromosomes and QTL detection for fiber quality and yield traits in RIL population.** All legends are same as described for **Figure 1**.

Among 63,058 SNPs used for screening RIL population, 3120 SNP markers (4.9%) were polymorphic between the two parents. Of those, 2618 were mapped on 26 chromosomes of upland cotton. The total length of this map was 1784.28 cM with average marker density of 0.68 cM (**Table 4**; Supplementary Table S1; and **Figures 1–5**). There were 101 SNPs on each chromosome averagely, with 1198 SNPs on At subgenome and 1420 SNPs on Dt subgenome, respectively. Uneven distribution of SNP markers on cotton chromosomes was observed. Chr14 had the highest number of SNPs (274 loci), while Chr12 had the lowest SNPs (15 loci). The average chromosome length was 68.63 cM, and the longest chromosome was Chr18 with 119.97 cM and the shortest one was Chr23 with 30.93 cM. The total lengths of At and Dt subgenomes were 888.61 and 902.67 cM, respectively. There were more loci on Dt subgenome than At subgenome. Twenty-two gaps (marker interval > 10 cM) were found on this genetic map, in which, 10 on At subgenome and 12 on Dt subgenome were observed.

**Table 4:** Summary of the high-density SNP map based on upland cotton RIL population.

Chromosome	Size (cM)	Mean distance (cM)	No SNP	Gap > 10 cM	Distortion ratio (%)^a^	SDR^b^
Chr01	52.90	0.40	133	0	15.79	2
Chr02	95.22	0.84	114	1	11.40	1
Chr03	116.96	1.72	68	3	27.94	3
Chr04	34.26	1.14	30	0	3.33	0
Chr05	69.80	0.52	135	0	7.41	0
Chr06	56.76	1.77	32	0	15.63	1
Chr07	67.03	0.96	70	0	15.71	1
Chr08	56.77	0.52	109	1	18.35	3
Chr09	99.90	0.62	162	1	9.26	1
Chr10	69.07	1.82	38	2	21.05	1
Chr11	41.46	0.86	48	0	18.75	1
Chr12	52.71	3.51	15	2	33.33	1
Chr13	68.76	0.28	244	0	12.70	3

At subgenome	881.61	0.74	1198	10	14.02	18

Chr14	86.01	0.31	274	0	1.46	0
Chr15	47.63	0.68	70	0	0.00	0
Chr16	74.37	0.43	172	1	10.47	2
Chr17	59.04	1.00	59	0	32.20	2
Chr18	119.97	1.02	118	2	23.73	2
Chr19	62.99	0.52	121	0	9.92	0
Chr20	80.10	0.90	89	2	21.35	3
Chr21	72.88	1.46	50	4	12.00	0
Chr22	50.24	0.72	70	0	8.57	1
Chr23	30.93	1.00	31	1	19.35	1
Chr24	73.43	0.34	219	0	7.76	2
Chr25	79.85	0.94	85	1	41.18	3
Chr26	65.21	1.05	62	1	16.13	1

Dt subgenome	902.67	0.64	1420	12	12.68	17

Total	1784.28	0.68	2618	22	13.29	35

*^a^The percentage of distortion loci account for the total loci each chromosome. ^b^SDR, segregation distorted region.*

### Segregation Distortion

Among the 2618 mapped SNPs, 13.29% (348) showed segregation distortion and most loci (71.26%) showed a higher allelic frequency from the female parent (**Table 4**; **Figures 1–5**). These SNPs were unevenly distributed on the 26 cotton chromosomes and ranged from 0 to 35 loci on each chromosome (**Table 4**). As previous report (Yu et al., 2011b), more distorted loci were located on the Dt subgenome than on the At subgenome (180 versus 168). Segregation distortion was non-random across the linkage map. Three chromosomesChr12, Chr17, and Chr25, showed serious segregation distortions of 33.33, 32.20, and 41.18%, respectively. Furthermore, a total of 35 SDRs were found on 20 chromosomes with 18 SDRs on the At subgenome and 17 SDRs on the Dt subgenome (**Table 4**; **Figures 1–5**). Interestingly, the distorted loci in some of the SDRs (SDR03-2, SDR06-1, SDR20-1, and SDR25-2) skewed toward the same allele and showed similar degree of segregation(**Figures 1–5**).

### Collinearity Analysis

**FIGURE 6:**
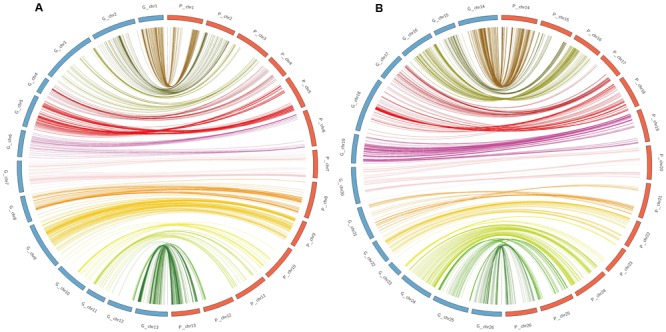
**Collinearity between the genetic map (blue) and the physical map (orange). (A)** Collinearity of the At subgenome between the genetic map and the physical map. **(B)** Collinearity of the Dt subgenome between the genetic map and the physical map.

All the mapped 2618 SNPs were aligned to the *G. hirsutum* reference genome to validate the genetic map. Alignments indicated that the genetic map constructed in the present study had good collinearity with the physical map (**Figure 6**), suggesting the high quality of the RIL map. However, several deviation on Chr04, Chr05, and Chr10 in the At subgenome and Chr16, Chr17, Chr18, Chr21, Chr23, and Chr25 in the Dt subgenome were detected between the genetic map and the physical map. The Dt subgenome showed good coverage of the physical map, representing 96.81% of the genome assembly length, while the At subgenome showed a lower coverage of 88.33% (Supplementary Table S2; **Figure 6**).

### QTL Analysis of Fiber and Yield Traits

A total of 71 QTLs for fiber quality and yield traits with 4.70∼32.28% of the total explained phenotypic variance (PV) were identified by CIM analysis (Supplementary Table S3; **Figures 1–5**). In these QTLs, 35 were positive additive indicating HS46 contributed alleles leading to an increase in relevant traits, while 36 were negative additive which meant MARCABUCAG8US-1-88 contributed alleles to increase fiber quality and yield traits. These QTLs were detected on 21 chromosomes, except Chr02, Chr06, Chr08, Chr13, and Chr26. Sixteen QTLs explaining 5.35∼32.28% of the PV were detected in both locations (**Table 5**). Among them, three were three QTLs each for FL, FU, MIC, FE, and LP, and one for BW.

**Table 5:** Stable quantitative trait locus (QTLs) for fiber quality and yield identified in the upland cotton RIL population.

Traits^a^	QTL	Environment^b^	Flanking markers	Position^c^	LOD^d^	Additive^e^	*R*^2^(%)^f^
FL	qFL-Chr10-1	Yc	i11502Gh and i33011Gh	44.51	2.65	0.29	6.30
		Bg	i11502Gh and i33011Gh	44.51	2.68	0.26	6.27
	qFL-Chr14-3	Yc	i15340Gh and i34657Gh	23.31	7.71	-0.46	15.05
		Bg	i34657Gh and i40518Gh	25.71	2.50	-0.27	6.21
	qFL-Chr15-1	Yc	i02955Gh and i02314Gh	13.11	3.69	-0.36	8.38
		Bg	i02955Gh and i02315Gh	12.31	5.21	-0.38	11.12
FU	qFU-Chr09-1	Yc	i50203Gb and i17373Gh	3.81	3.33	-0.24	7.73
		Bg	i50203Gb and i17373Gh	3.81	4.17	-0.29	10.94
	qFU-Chr09-2	Yc	i41596Gh and i26826Gh	47.11	3.12	-0.24	5.97
		Bg	i41596Gh and i26826Gh	47.11	2.68	-0.24	5.58
	qFU-Chr09-3	Yc	i06281Gh and i07773Gh	52.61	3.63	0.30	7.48
		Bg	i18943Gh and i07773Gh	52.61	2.76	0.29	6.31
MIC	qMIC-Chr14-1	Yc	i15340Gh and i34657Gh	23.31	2.51	0.08	5.52
		Bg	i15345Gh and i00465Gh	20.91	2.52	0.11	5.64
	qMIC-Chr16-1	Yc	i46435Gh and i62679Gt	51.01	2.89	0.08	6.53
		Bg	i01613Gh and i58367Gb	49.31	2.54	0.09	5.77
	qMIC-Chr16-2	Yc	i21384Gh and i22249Gh	57.01	3.36	0.09	7.35
		Bg	i44137Gh and i18258Gh	57.41	4.23	0.15	9.20
FE	qFE-Chr14-1	Yc	i15343Gh and i21369Gh	16.81	7.80	0.30	17.53
		Bg	i15343Gh and i21369Gh	15.71	7.42	0.26	15.15
	qFE-Chr20-1	Yc	i47006Gh and i17500Gh	41.51	3.28	0.20	7.40
		Bg	i17500Gh and i47439Gh	47.11	6.19	0.44	32.28
	qFE-Chr24-1	Yc	i04503Gh and i04704Gh	73.31	2.62	0.75	5.78
		Bg	i04503Gh and i04704Gh	73.31	2.49	0.62	5.35
BW	qBW-Chr10-1	Yc	i25267Gh and i30274Gh	32.21	2.82	0.61	27.96
		Bg	i25267Gh and i30274Gh	32.21	2.69	0.60	24.73
LP	qLP-Chr04-1	Yc	i20890Gh and i24786Gh	11.41	3.50	0.57	8.20
		Bg	i44575Gh and i24758Gh	11.11	3.28	0.56	7.49
	qLP-Chr10-1	Yc	i38146Gh and i22401Gh	35.11	4.20	-2.32	8.86
		Bg	i25267Gh and i30274Gh	33.21	2.68	-2.27	20.71
	qLP-Chr12-1	Yc	i40974Gh and i48211Gh	4.01	3.38	0.66	10.42
		Bg	i40974Gh and i48211Gh	4.01	2.81	0.64	11.79

*^a^FL, fiber length; FU, fiber uniformity; MIC, micronaire; FE, fiber elongation; FS, fiber strength; BW, boll weight; LP, lint percentage. ^b^Yc, Yacheng of Hainan Province; Bg, Baogang of Hainan Province. ^c^Position of QTL located on chromosome: as cM distance from the top of each chromosome. ^d^A LOD threshold of 2.5 was used for declaration of QTL, based on 1000 permutations at a significance level of 0.01. ^e^Positive “additive effect” indicates an increasing effect from HS46; negative “additive effect” indicates an increasing effect fromMARCABUCAG8US-1-88. ^f^Phenotypic variance explained by QTL.*

#### Fiber Length

Twelve QTLs were detected, explaining 5.28∼17.98% of the PV, and located on Chr05, Chr09, Chr10, Chr12, Chr14, Chr15, and Chr19 (Supplementary Table S3; **Figures 1–5**). Alleles that increased FL at 10 loci were derived from MARCABUCAG8US-1-88, whereas two positive alleles were contributed by HS46. Across both environments, qFL-Chr10-1, qFL-Chr14-3, and qFL-Chr15-1 were stably identified at marker intervals of i11502Gh-i33011Gh, i15340Gh-i40518Gh, and i02955Gh-i02315Gh, explaining 6.27∼6.30, 6.21∼15.05, and 8.38∼11.12% of the PV, respectively (**Table 5**).

#### Fiber Uniformity

A total of eight QTLs were mapped on Chr01, Chr05, Chr09, and Chr19 (Supplementary Table S3; **Figures 1–5**), which explained 5.28∼17.98% of the PV. Notably, all of the loci showed negative effects originating from MARCABUCAG8US-1-88. The details of three stable QTLs detected in all environments were as follows: qFU-Chr09-1, flanked by markers i50203Gb and i17373Gh, and explained 7.73∼10.94% of PV; qFU-Chr09-2, between markers i41596Gh and i26827Gh, accounting for 5.58∼5.97% of the FU variance; qFU-Chr09-3 located in the intervals between markers i06281Gh and i07773Gh, explaining 6.31∼7.48% of the PV. Interestingly, all these three stable QTLs were on Chr09 (**Table 5**).

#### Micronaire

Ten QTLs were identified and located on 8 chromosomes (Chr01, Chr05, Chr07, Chr10, Chr14, Chr16, Chr17, and Chr24), explaining 5.52∼18.24% of the PV (Supplementary Table S3; **Figures 1–5**). Alleles for increasing MIC at nine loci were contributed by HS46, and one loci was contributed by MARCABUCAG8US-1-88. qMIC-Chr14-1, qMIC-Chr16-1, and qMIC-Chr16-2, detected in both environments, were located in the intervals between i15345Gh and i34657Gh, i46435Gh and i62679Gt, and i21384Gh and i22249Gh, respectively. They explained 5.52∼5.64, 5.77∼6.53, and 7.35∼9.20% of the PV, accordingly (**Table 5**).

#### Fiber Elongation

Nine QTLs were mapped on Chr11, Chr14, Chr16, Chr17, Chr18, Chr20, and Chr24, explaining 5.65∼32.28% of the PV (Supplementary Table S3; **Figures 1–5**). All favorable QTL effects were contributed by HS46. Stable detection of qFE-Chr14-1, qFE-Chr20-1, and qFE-Chr24-1 flanked by SNP markers i15343Gh and i21369Gh, i47006Gh and i47439Gh, i04503Gh and i04704Gh were in both environments, explained15.15∼17.53, 7.40∼32.28, and 5.35∼5.78% of the FE variance, respectively (**Table 5**). Three major QTLs, qFE-Chr14-1, qFE-Chr14-3, and qFE-Chr20-1, explained more than 10% of variation.

#### Fiber Strength

Eight QTLs, explaining 5.14∼9.96% of the total PV, were detected on Chr05, Chr14, Chr19, and Chr20 (Supplementary Table S3; **Figures 1–5**). Alleles for increasing FS on Chr05 and Chr19 were contributed by HS46, and positive alleles on Chr14 and Chr20 came from MARCABUCAG8US-1-88. All of these eight QTLs were detected in single environment, indicating that the environmental effects were important for the performance of FS.

#### Boll Weight

For BW, among eight identified QTLs, seven were located on six chromosomes (Chr10, Chr16, Chr18, Chr23, Chr24, and Chr25) and the remaining two on Chr09 (Supplementary Table S3; **Figures 1–5**). Alleles for increasing BW at the loci on Chr09, Chr10, Chr18, Chr23, and Chr24, were contributed by HS46, and on Chr16 was from MARCABUCAG8US-1-88. All other seven QTLs were identified only in one environment (**Table 5**), with the exception of qBW-Chr10-1 which was identified in both environments. Four major QTLs, qBW-Chr09-2, qBW-Chr10-1, qBW-Chr18-1, and qBW-Chr23-1, explaining 10.62%, 24.73%/27.96% (detected in two environments), 12.28, and 23.55% of the PV, respectively, were important in the improvement of BW.

#### Lint Percent

Fifteen QTLs were detected on Chr03, Chr04, Chr05, Chr10, Chr12, Chr14, Chr16, Chr17, Chr20, Chr21, and Chr22 (Supplementary Table S3; **Figures 1–5**). Among the 15 non-over lapping QTLs, qLP-Chr04-1, qLP-Chr10-1, and qLP-Chr12-1 were detected in both environments. Four major QTLs, qLP-Chr10-1, qLP-Chr12-1, qLP-Chr14-1, and qLP-Chr17-1, explained more than 10% of variation. Among them, alleles increasing LP at Chr10, Chr14, and Chr17 came from MARCABUCAG8US-1-88, whereas the one on Chr12 was derived from HS46.

### QTL Clusters and Hotspots

Quantitative trait locus were not randomly distributed across chromosomes and chromosomal regions. Some QTLs were identified as “cluster” and “hotspot,” where clusters and hotspots were defined to contain multiple QTLs within 20 cM regions, approximately, for different and same traits, respectively (Guo et al., 2007; Rong et al., 2007; Said et al., 2013).

In the current study, there were two QTL clusters on Chr05 which contained three and five QTLs, respectively (**Table 6**). The Chr05-cluster-1, which possessed three QTLs, was found at 11∼13 cM for FL, MIC, and LP and Chr05-cluster-2 with five QTLs was located at 40∼55 cM for FL, FU, and FS. The FL hotspot, Chr05-hotspot-1, carrying three QTLs, was located at 40∼53 cM. It should be noted that the position of the hotspot coincided with the second identified cluster.

**Table 6:** Quantitative trait locus Cluster/Hotspot for fiber quality and yield in the upland cotton RIL population across two environments.

Cluster/Hotspot^a^	Location^b^	QTL
Chr05-cluster-1	11–13 cM	qFL-Chr05-3, qLP-Chr05-1, qMIC-Chr05-1
Chr05-cluster-2	40–55 cM	qFL-Chr05-4, qFL-Chr05-1, qFU-Chr05-1, qFL-Chr05-2, qFS-Chr05-1
Chr05-hotspot-1	40–53 cM	qFL-Chr05-4, qFL-Chr05-1, qFL-Chr05-2
Chr09-cluster-1	46–62 cM	qBW-Chr09-1, qFU-Chr09-2, qFU-Chr09-3, qFU-Chr09-4
Chr09-hotspot-1	47–62 cM	qFU-Chr09-2, qFU-Chr09-3, qFU-Chr09-4
Chr10-cluster-1	32–45 cM	qBW-Chr10-1, qLP-Chr10-1, qLP-Chr10-2, qFL-Chr10-1
Chr14-cluster-1	6–26 cM	qFS-Chr14-1, qFL-Chr14-1, qFS-Chr14-2, qFE-Chr14-1, qFL-Chr14-2, qFS-Chr14-3, qFL-Chr14-3, qMIC-Chr14-1
Chr14-hotspot-1	14–26 cM	qFL-Chr14-1, qFL-Chr14-2, qFL-Chr14-3
Chr14-hotspot-2	6–22 cM	qFS-Chr14-1, qFS-Chr14-2, qFS-Chr14-3
Chr14-hotspot-3	1–17 cM	qFE-Chr14-2, qFE-Chr14-3, qFE-Chr14-1
Chr19-cluster-1	20–28 cM	qFU-Chr19-1, qFL-Chr19-1, qFS-Chr19-1, qFS-Chr19-2
Chr20-cluster-1	41–60 cM	qFE-Chr20-1, qFS-Chr20-1, qFS-Chr20-2

*^a^Cluster, a number of QTLs for different traits within approximately 20 cM regions; Hotspot, a multiple QTLs for the same traits within a 20 cM region. ^b^Position of QTL Cluster/Hotspot located on chromosome: as cM distance from the top of each chromosome.*

Chr09 contained one cluster and one hotspot (**Table 6**). The Chr09-cluster-1, identified at 46∼62 cM, carried four QTLs for FU and BW while Chr09-hotspot-1, identified at 47∼62 cM, carried three QTLs of FU and overlapped the cluster Chr09-cluster-1.

Chr10 contained one cluster (Chr10-cluster-1) pertaining to FL, BW, and LP QTLs (**Table 6**). This cluster was located at 32∼45 cM, containing four QTLs.

Chr14 contained one cluster and three hotspots (**Table 6**). The Chr14-cluster-1 was located at 6∼26 cM and carried eight QTLs for FL, FS, MIC, and FE. The two hotspots, Chr14-hotspot-1 and Chr14-hotspot-2, identified fell into the range of the Chr14-cluster-1, and were FL and FS related hotspots, respectively. Furthermore, the three hotspots Chr14-hotspot-1, Chr14-hotspot-2, and Chr14-hotspot-3 for FE, FS, and FL overlapped at 1∼17, 6∼22, and 14∼26 cM, respectively, indicating a possible fiber quality hotspot cluster in the region.

Chr19 contained one cluster, Chr19-cluster-1, which ranged from 20∼28 cM, and carried four QTLs for FL, FU, and FS (**Table 6**).

Chr20 had one cluster Chr20-cluster-1, which was identified at 41∼60 cM and carried three QTLs related to FE and FS(**Table 6**).

### Identification of the Epistatic and QTL × Environment Interactions Loci

**FIGURE 7:**
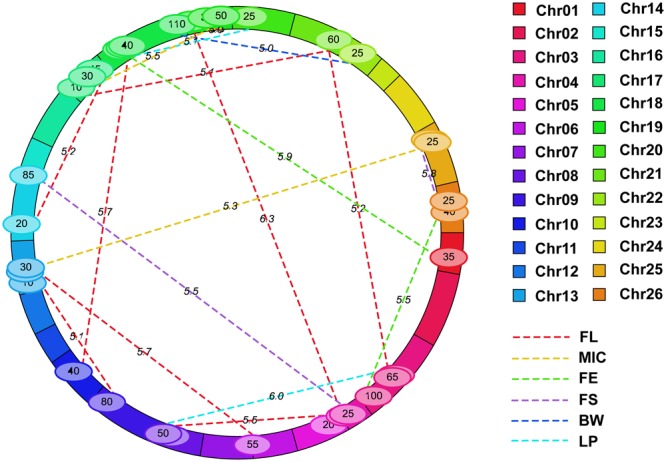
**Epistatic and QE interaction loci for fiber quality and yield of RILs across two environments by IciMapping.** The solid bars with different colors represent different chromosomes and the dotted line with different colors indicates different chromosomes, for which the legend is given at the side of figure. FL, fiber length; FU, fiber uniformity; MIC, micronaire; FE, fiber elongation; FS, fiber strength; BW, boll weight; LP, lint percentage.

Nineteen e-QTLs were identified by the MET analysis of the multi-environment phenotypic values (Supplementary Table S4; **Figure 7**). Fifteen of them had both an epistatic main effect and minor epistasis × environment interaction effect while remaining four had the former effect only. The explained PV by each QTL ranged from 3.89 to 6.22%, while that by each QTL × environment interaction only ranged from 0.0 to 0.67%. Among them, the highest number of e-QTLs (9) was detected for FL, three for MIC, two for FE, two for FS, one for BW, and two for LP. No e-QTL was detected for FU. For FL, there were two loci i38186Gh-i11502Gh and i15345Gh-i18849Gh overlapped with two additive QTLs, qFL-Chr10-1 and qFL-Chr14-2, indicating both additive and epistatic value of these two loci had played important role in FL. One pair of interacting marker intervals, i08786Gh-i00558Gh and i09371Gh-i09643Gh, was detected for FL on the same chromosome of Chr19, whereas other interacted loci were located on different chromosomes. In addition, some marker intervals had interactions with other multiple marker intervals to control same or different traits. The marker interval, i22642Gh-i41613Gh on Chr21, had interaction with two marker intervals, i35903Gh-i20966Gh on Chr03 and i44474Gh-i03341Gh on Chr17, for FL. The marker interval i16566Gh-i08941Gh on Chr19 interacted with two marker intervals, i14920Gh-i51624Gb on Chr17 and i08832Gh-i09452Gh on Chr19, for MIC. The marker interval i10502Gh-i36496Gh on Chr04 had interactions with two marker intervals, i08933Gh-i28797Gh on Chr19 and i05035Gh-i22015Gh on Chr14, to control two traits, FL and FS, respectively. The marker interval i32883Gh-i13851Gh on Chr18 had interactions with two marker intervals including i38186Gh-i11502Gh on Chr10 and i02298Gh-i42430Gh on Chr01 pertaining to FL and FE, respectively.

## Discussion

### SNP Discovery and map Construction

With the development of theoretical and applied genetic breeding research, high-density genetic maps are becoming more and more important. SNPs were proved to be the most abundant form of genetic variation, providing a rich source of DNA markers (Agarwal et al., 2008). Recently, SNP arrays for many crops had been developed and utilized in MAS breeding (Fang et al., 2013; Gao et al., 2015; Wang X. et al., 2015). In present work, 2618 polymorphic SNP markers from a 63K SNP assay (Hulse-Kemp et al., 2015) were used to construct a relative high-density genetic map for 188 RILs derived from the combination of HS46/MARCABUCAG8US-1-88.

The total length of the linkage map was 1784.28 cM, far longer than previously map lengths reported with the same cross (Shappley et al., 1998; Wu et al., 2008). The interval of the map was 0.68 cM/marker, increasing the density of the previous linkage map to the thickest linkage map in upland cotton and representing a considerable advance over previously map researches based on RFLP, SSR, AFLP, ITISJ, SRAPetc (Shappley et al., 1998; Ulloa et al., 2002; Shen et al., 2005, 2006; Zhang et al., 2005, 2009; Wu et al., 2008; Tan et al., 2014; Liu et al., 2015). The collinearity analysis showed that the constructed map had good collinearity with the *G. hirsutum* reference genome, indicating the high quality and accuracy of the map. One main reason for the lower coverage of At subgenome than Dt subgenome was that two linkage groups, Chr12 and Chr10, only represent 10.91 and 70.34% of the corresponding chromosomes, respectively.

Consistent with previous studies (Tan et al., 2014; Cao et al., 2015; Hulse-Kemp et al., 2015), more markers were found on Dt subgenome (54.24%) than At subgenome (45.76%) in the present map, which was attributed to the lower level of polymorphism in At subgenome of upland cotton. The Dt subgenome was longer than At subgenome in this map, duo to Dt subgenome with more loci experienced a higher frequency of recombination (Guo et al., 2007). Although the average density was high, there were still some gaps in several chromosomes. Moreover, like previous maps (Shen et al., 2006; Tan et al., 2014; Cao et al., 2015), although the markers of our map distributed evenly on the entire genome, there were still some chromosomes anchored more markers than others, which might be attributed to the non-random distribution of markers and the lack of marker polymorphism between mapping parents on some chromosomes because of relative narrow range of genetic diversity in upland cotton.

Segregation distortion, regarded as the important source of plant evolution, widely exists in plant populations. The species, population types, crosses, and marker types of plants will lead to significant variance in segregation distortion with different origin, genetic effects, and degree (Xu et al., 1997). The reports from intraspecific population of upland cotton indicated that the ratio of segregation distortion enhanced with the increase of divergence level of the parents (Shappley et al., 1998; Ulloa et al., 2002; Shen et al., 2005, 2006), and the ratio of segregation distortion in the RIL population was higher than that in the F_2_ population, which might mainly result from genetic drift (Shen et al., 2006). In present study, 13.29% of the total loci showed segregation distortion (*P* < 0.05), which was similar to previous maps of upland cotton (16.6∼36.7%; Tan et al., 2014; Cao et al., 2015; Liu et al., 2015). Most distorted loci skewed to HS46 alleles, which might be the result of chromosomal elements markers diverged via female drive in the meiosis (Fishman and Willis, 2005). Furthermore, most segregation distortion loci occurred in the clusters, like previously reported for interspecific and intraspecific populations (Guo et al., 2007; Zhang et al., 2009; Yu et al., 2011b; Tan et al., 2014; Liu et al., 2015). The loci clustering on the same chromosome or within the same SDR skewed to the same allele suggested that genetic hitchhiking effects existed in upland cotton (Yu et al., 2011b).

### Significance and Potential Application of QTL Mapping

The genotypic value for HS46 was greater (*P* < 0.05) than that for MARCABUCAG8US-1-88 with respect to FL, FU, BW, FS, and MIC. Similar to the same cross used by Wu et al. (2008), no significant difference between the two parents was detected for FE and LP. Even though the two parents were phenotypically similar regarding these two traits, due to genetic dissimilarities between the two parents, significant differences in this RIL population existed. In the present research, FS had a low heritability and easily affected by environment. Similar results could be found in previous literatures about a four-way cross population in upland cotton (Qin et al., 2008). The obvious difference indicated FS was highly influenced by the experimental environment and difficult in genetic improvement. Wang et al. (2015a) suggested FS was a moderate heritability trait and that all the fiber quality and yield component traits presented significant environmental effects. Moreover, in the research based on same population, FS did not show a higher heritability in all the measured traits but a significant *VGE/VP* instead (Wu et al., 2008). Therefore, the heritability of FS was not stable and affected by different population and environment.

Similarly, BW was highly influenced by environments. Furthermore, BW was significantly and positively correlated with MIC and FS. Consequently, it is feasible to improve BW by selecting these correlated traits, having more accurate repeatability across environments in breeding. Stable QTLs such as qFL-Chr14-3, qFL-Chr15-1, qFU-Chr09-1, qFE-Chr14-1 qFE-Chr20-1, qBW-Chr10-1, qLP-Chr10-1, and qLP-Chr12-1 could be used in breeding. It was more likely that these QTLs could be used to identify candidate genes for these related traits because of the availability of high-density SNP markers. In such case, they will be the potential candidates for fine mapping and ultimate candidate gene discovery.

Although there were many differences in parental lines, mapping populations, and markers type, our results were comparable with earlier identified QTLs. Searching for QTLs of upland cotton in a CottonQTLdb database^3^ developed by Said et al. (2015) and statistical data on cotton QTL previously, there were seven QTLs for FL, two QTLs for FU, two QTLs for MIC, four QTLs for FE, two QTLs for FS, and five QTLs for LP in present work, sharing same genetic position (spacing distance < 5 cM) and physical position with earlier reports (Wu et al., 2008; Tan et al., 2014; Liu et al., 2015; Zhang Z. et al., 2015). In addition, five QTLs for FL, six QTLs for FU, eight QTLs for MIC, four QTLs for FE, seven QTLs for FS, six QTLs for BW, and seven QTLs for LP had been mapped on the same chromosomes but not on the same position as reported in the previous researches. These inconsistencies might be due to different genetic backgrounds and DNA markers (Shappley et al., 1998; Wu et al., 2008; Said et al., 2015). Remaining nine additive QTLs (qFE-Chr17-1, qBW-Chr09-1, qBW-Chr09-2, qBW-Chr23-1, qBW-Chr24-1, qBW-Chr25-1, qLP-Chr20-1, qLP-Chr21-1, and qLP-Chr21-2) might be novel loci, due to unavailability of any reports for these traits on those chromosomes. As the number of identified QTLs for fiber quality and yield traits increased, the genetic control of fiber quality and yield will be better understood.

### Molecular Mechanism of Trait Correlation and Linkage Drag

Co-localization of QTLs on chromosomes, referred to as “QTL cluster/hotspot,” has previously been reported in cotton (Shappley et al., 1998; Qin et al., 2008; Said et al., 2013) and many other species (Gonzalez et al., 2015; Li et al., 2015). In the present study, 12 certain genomic regions, especially, Chr05, Chr09, Chr10, Chr14, Chr19, and Chr20, were investigated for their involvement in controlling one (hotspot) or more (clusters) fiber quality or yield traits, the similar result was also reported in the publications (Qin et al., 2008; Sun et al., 2011; Said et al., 2013; Yu et al., 2013). The existence of QTL clusters explained why so many traits were highly interrelated.

Based on the comprehensive analysis of clusters and hotspots in this study, breeding programs targeting fiber quality or yield traits can focus on hotspot clustering regions and select around the region. Notably, almost all the hotspots overlapped QTL clusters. The presence of QTL clusters and hotspots proved that genes related to certain traits were more heavily concentrated in certain regions of genome than others (Said et al., 2013). The discovery of cluster and hotspot may be useful in MAS breeding program and may help breeders to select the traits of interests and find novel QTLs once the markers have anchored these regions.

### Interaction between Loci within and Across Chromosomes

It was successful to identify e-QTLs with both additive and epistatic effects, e-QTL pairs and epistasis × environment interactions in the present work, which were often neglected in some complex trait studies. Generally, if the proportion of PV explained by the identified additive QTL is close to broad-sense heritability, epistasis is less important (Li et al., 2007). However, for FL, the total PV explained by additive QTLs were much lower than the broad-sense heritability (66%, **Table 2**), indicating that there were epistatic interactions in these loci. Finally, nine pairs of e-QTLs for FL were detected in our work, of which there were two loci, i38186Gh-i11502Gh on Chr10 and i15345Gh-i18849Gh on Chr14, locating on the same position with two additive QTLs (qFL-Chr10-1 and qFL-Chr14-2), suggesting that both addictive and epistatic effects had played important roles in genetic control of FL. In fact, interactions among loci or QTL × environmental factors contributed a substantial effect to complex trait phenotypic variation (Carlborg and Haley, 2004). Several novel QTLs and specific trait relationships between loci, within and across chromosomes, could be considered as the interactions between loci (Grosse-Brinkhaus et al., 2010).

## Conclusion

A high-density linkage map was constructed in the upland cotton RIL population using the 63K cotton SNP array. Nine novel QTLs, seven pleiotropic QTL clusters, five hotspots, and 19 e-QTLs for fiber quality and yield traits were identified with tightly linked SNP markers. These QTLs could serve as target regions for map-based gene cloning and MAS in cotton breeding.

## Author Contributions

CoL and SZ designed and conducted the experiments, analyzed data and wrote the manuscript. CoL and YD performed phenotyping and data analysis, CoL, TZ, and LL participated in field trials. ChL, EY, LM, QH, and MD prepared and reviewed the manuscript. SZ and JC designed and supervised the experiments. All authors have read and approved the final manuscript.

## Conflict of Interest Statement

The authors declare that the research was conducted in the absence of any commercial or financial relationships that could be construed as a potential conflict of interest.

## Footnotes

1 http://ibi.zju.edu.cn/software/qtmodel/index.html

2 http://www.emtd.com.cn/

3 http://www.cottonqtldb.org
