# Supplementary material for: Genome-Wide SNP Linkage Mapping and QTL Analysis for Fiber Quality and Yield Traits in the Upland Cotton Recombinant Inbred Lines Population
Source: Front Plant Sci. 2016 Sep 8;7:1356. doi: 10.3389/fpls.2016.01356 (PMC5014859; doi:10.3389/fpls.2016.01356)
Supplement: Supplementary file 3 [file Table_3.DOC]

**Table S3 QTLs for fiber quality and yield in the upland cotton RIL population in different environments**

| **Traits^a^** | **QTL** | **Environment^b^** | **Flanking markers** | **Position^c^** | **LOD^d^** | **Additive^e^** | **R^2^(%)^f^** |
| --- | --- | --- | --- | --- | --- | --- | --- |
| FL | qFL-Chr05-1 | yc | i29825Gh and i38533Gh | 45.31 | 3.86 | -0.37 | 8.61 |
|  | qFL-Chr05-2 | yc | i09095Gh and i46446Gh | 52.11 | 3.27 | -0.33 | 7.75 |
|  | qFL-Chr05-3 | bg | i17756Gh and i21516Gh | 11.21 | 2.60 | -1.16 | 5.28 |
|  | qFL-Chr05-4 | bg | i12470Gh and i45534Gh | 40.01 | 3.51 | -0.32 | 7.63 |
|  | qFL-Chr09-1 | bg | i50203Gb and i17373Gh | 3.81 | 3.36 | -0.30 | 8.11 |
|  | qFL-Chr10-1 | yc | i11502Gh and i33011Gh | 44.51 | 2.65 | 0.29 | 6.30 |
|  |  | bg | i11502Gh and i33011Gh | 44.51 | 2.68 | 0.26 | 6.27 |
|  | qFL-Chr12-1 | yc | i40974Gh and i08075Gh | 16.21 | 3.07 | -0.33 | 7.38 |
|  | qFL-Chr14-1 | yc | i42774Gh and i31037Gh | 14.21 | 9.36 | -0.50 | 17.98 |
|  | qFL-Chr14-2 | yc | i15345Gh and i00465Gh | 20.91 | 5.51 | -0.41 | 11.20 |
|  | qFL-Chr14-3 | yc | i15340Gh and i34657Gh | 23.31 | 7.71 | -0.46 | 15.05 |
|  |  | bg | i34657Gh and i40518Gh | 25.71 | 2.50 | -0.27 | 6.21 |
|  | qFL-Chr15-1 | yc | i02955Gh and i02314Gh | 13.11 | 3.69 | -0.36 | 8.38 |
|  |  | bg | i02955Gh and i02315Gh | 12.31 | 5.21 | -0.38 | 11.12 |
|  | qFL-Chr19-1 | yc | i24482Gh and i50703Gb | 22.51 | 3.77 | 0.46 | 7.87 |
| FU | qFU-Chr01-1 | bg | i02201Gh and i27043Gh | 14.51 | 2.86 | -0.61 | 8.77 |
|  | qFU-Chr05-1 | bg | i29825Gh and i19536Gh | 45.31 | 2.71 | -0.22 | 6.41 |
|  | qFU-Chr09-1 | yc | i50203Gb and i17373Gh | 3.81 | 3.33 | -0.24 | 7.73 |
|  |  | bg | i50203Gb and i17373Gh | 3.81 | 4.17 | -0.29 | 10.94 |
|  | qFU-Chr09-2 | yc | i41596Gh and i26826Gh | 47.11 | 3.12 | -0.24 | 5.97 |
|  |  | bg | i41596Gh and i26827Gh | 47.11 | 2.68 | -0.24 | 5.58 |
|  | qFU-Chr09-3 | yc | i06281Gh and i07773Gh | 52.61 | 3.63 | -0.30 | 7.48 |
|  |  | bg | i18943Gh and i07773Gh | 52.61 | 2.76 | -0.29 | 6.31 |
|  | qFU-Chr09-4 | yc | i05874Gh and i04639Gh | 61.41 | 2.52 | -0.20 | 5.00 |
|  | qFU-Chr09-5 | bg | i44757Gh and i33003Gh | 18.11 | 2.56 | -0.24 | 4.70 |
|  | qFU-Chr19-1 | yc | i09219Gh and i09217Gh | 20.81 | 3.06 | -0.22 | 6.79 |
| MIC | qMIC-Chr01-1 | yc | i14520Gh and i27043Gh | 14.51 | 2.55 | 0.24 | 9.29 |
|  | qMIC-Chr05-1 | yc | i23000Gh and i46433Gh | 12.61 | 2.72 | -0.25 | 18.24 |
|  | qMIC-Chr07-1 | bg | i01629Gh and i01824Gh | 59.31 | 2.67 | 0.10 | 5.89 |
|  | qMIC-Chr10-1 | yc | i00538Gh and i22107Gh | 62.61 | 3.50 | 0.16 | 9.08 |
|  | qMIC-Chr14-1 | yc | i15340Gh and i34657Gh | 23.31 | 2.51 | 0.08 | 5.52 |
|  |  | bg | i15345Gh and i00465Gh | 20.91 | 2.52 | 0.11 | 5.64 |
|  | qMIC-Chr16-1 | yc | i46435Gh and i62679Gt | 51.01 | 2.89 | 0.08 | 6.53 |
|  |  | bg | i01613Gh and i58367Gb | 49.31 | 2.54 | 0.09 | 5.77 |
|  | qMIC-Chr16-2 | yc | i21384Gh and i22249Gh | 57.01 | 3.36 | 0.09 | 7.35 |
|  |  | bg | i44137Gh and i18258Gh | 57.41 | 4.23 | 0.15 | 9.20 |
|  | qMIC-Chr17-1 | bg | i28727Gh and i14844Gh | 44.81 | 3.16 | 0.12 | 7.12 |
|  | qMIC-Chr24-1 | bg | i04568Gh and i41753Gh | 16.81 | 3.25 | 0.11 | 6.98 |
|  | qMIC-Chr24-2 | bg | i04503Gh and i04704Gh | 73.31 | 3.73 | 0.44 | 7.69 |
| FE | qFE-Chr11-1 | bg | i33855Gh and i43823Gh | 5.31 | 2.78 | 1.16 | 6.60 |
|  | qFE-Chr14-1 | yc | i15343Gh and i21369Gh | 16.81 | 7.80 | 0.30 | 17.53 |
|  |  | bg | i15343Gh and i21369Gh | 15.71 | 7.42 | 0.26 | 15.15 |
|  | qFE-Chr14-2 | bg | i15536Gh and i05487Gh | 1.11 | 3.04 | 1.09 | 5.72 |
|  | qFE-Chr14-3 | bg | i22641Gh and i48509Gh | 6.31 | 3.86 | 0.25 | 12.99 |
|  | qFE-Chr16-1 | bg | i26628Gh and i22455Gh | 1.11 | 2.31 | 1.17 | 6.66 |
|  | qFE-Chr17-1 | bg | i18575Gh and i03527Gh | 42.31 | 3.12 | 0.18 | 7.19 |
|  | qFE-Chr18-1 | bg | i37702Gh and i13705Gh | 57.51 | 2.61 | 0.76 | 5.65 |
|  | qFE-Chr20-1 | yc | i47006Gh and i17500Gh | 41.51 | 3.28 | 0.20 | 7.40 |
|  |  | bg | i17500Gh and i47439Gh | 47.11 | 6.19 | 0.44 | 32.28 |
|  | qFE-Chr24-1 | yc | i04503Gh and i04705Gh | 73.31 | 2.62 | 0.75 | 5.78 |
|  |  | bg | i04503Gh and i04706Gh | 73.31 | 2.49 | 0.62 | 5.35 |
| FS | qFS-Chr05-1 | bg | i51309Gb and i16608Gh | 54.81 | 3.88 | 0.52 | 8.39 |
|  | qFS-Chr14-1 | yc | i22641Gh and i00458Gh | 6.71 | 2.53 | -0.21 | 5.14 |
|  | qFS-Chr14-2 | yc | i42774Gh and i31037Gh | 14.21 | 5.07 | -0.28 | 9.96 |
|  | qFS-Chr14-3 | yc | i25374Gh and i31263Gh | 21.61 | 4.11 | -0.26 | 8.15 |
|  | qFS-Chr14-4 | yc | i22394Gh and i41891Gh | 40.11 | 2.69 | -0.20 | 5.49 |
|  | qFS-Chr19-1 | bg | i09217Gh and i50703Gb | 22.51 | 3.35 | 0.51 | 7.82 |
|  | qFS-Chr19-2 | bg | i08973Gh and i08969Gh | 27.81 | 2.76 | 0.61 | 6.38 |
|  | qFS-Chr20-1 | yc | i17505Gh and i47439Gh | 42.11 | 3.53 | -0.50 | 8.13 |
|  | qFS-Chr20-2 | yc | i11551Gh and i11478Gh | 59.61 | 3.31 | -0.48 | 7.66 |
| BW | qBW-Chr09-1 | yc | i46552Gh and i36372Gh | 46.11 | 3.28 | 0.16 | 7.35 |
|  | qBW-Chr09-2 | bg | i19084Gh and i06193Gh | 32.31 | 2.73 | 1.29 | 10.62 |
|  | qBW-Chr10-1 | yc | i25267Gh and i30274Gh | 32.21 | 2.82 | 0.61 | 27.96 |
|  |  | bg | i25267Gh and i30274Gh | 32.21 | 2.69 | 0.60 | 24.73 |
|  | qBW-Chr16-1 | yc | i41790Gh and i01669Gh | 24.11 | 2.51 | -0.14 | 5.70 |
|  | qBW-Chr18-1 | yc | i64918Gm and i45991Gh | 87.21 | 3.56 | 1.56 | 12.28 |
|  | qBW-Chr23-1 | yc | i06287Gh and i06175Gh | 24.01 | 3.83 | 0.98 | 23.55 |
|  | qBW-Chr24-1 | bg | i28524Gh and i04688Gh | 34.41 | 3.32 | 0.22 | 9.30 |
|  | qBW-Chr25-1 | yc | i11287Gh and i10628Gh | 57.51 | 2.54 | 0.14 | 5.75 |
| LP | qLP-Chr03-1 | yc | i42939Gh and i34191Gh | 96.91 | 2.56 | 0.47 | 5.66 |
|  | qLP-Chr04-1 | yc | i20890Gh and i24786Gh | 11.41 | 3.50 | 0.57 | 8.20 |
|  |  | bg | i44575Gh and i24758Gh | 11.11 | 3.28 | 0.56 | 7.49 |
|  | qLP-Chr05-1 | yc | i45777Gh and i21516Gh | 11.21 | 2.94 | -2.43 | 6.56 |
|  | qLP-Chr10-1 | yc | i38146Gh and i22401Gh | 35.11 | 4.20 | -2.32 | 8.86 |
|  |  | bg | i25267Gh and i30274Gh | 33.21 | 2.68 | -2.27 | 20.71 |
|  | qLP-Chr10-2 | yc | i32686Gh and i00413Gh | 36.51 | 3.25 | -2.50 | 6.93 |
|  | qLP-Chr12-1 | yc | i40974Gh and i48211Gh | 4.01 | 3.38 | 0.66 | 10.42 |
|  |  | bg | i40974Gh and i48211Gh | 4.01 | 2.81 | 0.64 | 11.79 |
|  | qLP-Chr12-2 | yc | i25940Gh and i52799Gb | 52.01 | 2.62 | -2.26 | 5.73 |
|  | qLP-Chr14-1 | bg | i05711Gh and i43468Gh | 9.31 | 4.10 | -2.25 | 20.87 |
|  | qLP-Chr14-2 | bg | i05772Gh and i32398Gh | 69.31 | 2.61 | -0.61 | 6.02 |
|  | qLP-Chr16-1 | yc | i01572Gh and i43060Gh | 3.51 | 3.33 | -3.58 | 7.10 |
|  | qLP-Chr17-1 | bg | i60898Gt and i03341Gh | 7.51 | 3.17 | -2.98 | 12.66 |
|  | qLP-Chr20-1 | yc | i11535Gh and i39228Gh | 6.31 | 3.72 | 0.55 | 8.12 |
|  | qLP-Chr21-1 | yc | i00171Gh and i07540Gh | 2.01 | 2.50 | -2.20 | 5.35 |
|  | qLP-Chr21-2 | bg | i31481Gh and i35971Gh | 49.31 | 2.70 | 0.64 | 6.17 |
|  | qLP-Chr22-1 | yc | i45635Gh and i12927Gh | 16.61 | 2.72 | -2.40 | 6.37 |

^a^ FL: fiber length; FU: fiber uniformity; MIC: micronaire; FE: fiber elongation; FS: fiber strength; BW: boll weight; LP: lint percentage

^b^ yc: Yacheng of Hainan Province; bg: Baogang of Hainan Province

^c^ Position of QTL located on chromosome: as cM distance from the top of each chromosome

^d^ A LOD threshold of 2.5 was used for declaration of QTL, based on 1000 permutations at as significance level of 0.01

^e^ Positive “additive effect” indicates an increasing effect from HS46; negative “additive effect ”indicates an increasing effect from MARCABUCAG8US-1-88

^f^ Phenotypic variance explained by QTL
